# Personality variables among sexual offenders with and without diagnosis of paraphilic disorders

**DOI:** 10.1192/j.eurpsy.2021.1020

**Published:** 2021-08-13

**Authors:** W. Oronowicz-Jaśkowiak, M. Lew-Starowicz

**Affiliations:** 1 Iii Department Of Psychiatry, Instutute of Psychiatry and Neurology, Warsaw, Poland; 2 Department Of Psychiatry, CMKP, Warsaw, Poland

**Keywords:** paraphilic disorder, Big Five personality traits

## Abstract

**Introduction:**

Sexual offenders are classified in terms of the act they have committed, diagnosis of sexual preference disorder (paraphilic disorder), and the potential motives behind the act. The typology that is often used in forensic-sexological practice is the division into preferential and non-preferential perpetrators, i.e. perpetrators showing or not showing a sexual preference disorder.

**Objectives:**

The aim of the study was to assess whether psychosocial and personality variables significantly differ between the group of preferential and non-preferential sexual offenders.

**Methods:**

The study involved 120 persons, including 60 preferential and 60 non-preferential sexual offenders. The participants were presented with selected, standardized psychological tools to personality traits, self-esteem, life satisfaction, capacity to understand emotions, attachment style.

**Results:**

The study involved 120 persons, including 60 preferential and 60 non-preferential sexual offenders. The participants were presented with selected, standardized psychological tools to personality traits, self-esteem, life satisfaction, capacity to understand emotions, attachment style.

**Conclusions:**

Differences between the both study groups and the male standardization sample suggest worse psychosocial functioning of sexual offenders. A critical analysis of the methodological limitations of this study have been presented.

**Conflict of interest:**

Scientific work was financed from the budget for science in the years 2017-2021, as a research project DI 16/003046 under the programme „Diamond Grant”.

